# From albuminuria to multi-omics signatures: emerging biomarkers and drug targets for early-stage chronic kidney disease

**DOI:** 10.3389/fphar.2026.1765974

**Published:** 2026-04-09

**Authors:** Kaili Chen, Yadong Zheng, Siyu Huang, Linqi Zhang

**Affiliations:** 1 Department of Nephropathy, The First Affiliated Hospital of Henan University of Chinese Medicine, Zhengzhou, China; 2 First Clinical Medical College, Henan University of Chinese Medicine, Zhengzhou, China; 3 Institute of Rehabilitation Medicine, Henan Academy of Innovations in Medical Science, Zhengzhou, China

**Keywords:** albuminuria, biomarkers, chronic kidney disease, early-stage CKD, multi-omics, urinary proteomics

## Abstract

Chronic kidney disease (CKD) is highly prevalent worldwide, yet structural kidney injury often develops long before changes in estimated glomerular filtration rate (eGFR) or persistent albuminuria are detected. Albuminuria is a well-established prognostic marker and treatment target, but it captures only part of the biological diversity of early kidney damage, shows substantial within-person variability, and may miss risk in predominantly tubulointerstitial or microvascular forms of disease. Recent advances in high-throughput omics now allow detailed profiling of renal stress in urine, blood and tissue, yielding proteomic, metabolomic, transcriptomic, epigenetic and non-coding RNA signatures linked to tubular injury, inflammation, fibrosis, mitochondrial dysfunction and disturbed energy metabolism. When these molecular layers are analysed in combination, multi-omics signatures can improve risk stratification beyond conventional Kidney Disease: Improving Global Outcomes (KDIGO) staging, help to define mechanistically distinct patient subgroups and highlight candidate therapeutic targets in haemodynamic–metabolic, immune/complement and extracellular matrix pathways. In this mini-review, we summarise the emerging evidence supporting a move beyond an albuminuria-centred view of early CKD towards mechanistically informed, multi-omics-based biomarkers, and we outline key requirements for clinical translation, including analytical standardisation, longitudinal validation and proof that such markers deliver actionable gains in patient care.

## 1 Introduction

CKD is a major global health burden, affecting roughly one in ten adults and substantially increasing premature mortality and cardiovascular complications (GBD, 2023 Chronic Kidney Disease Collaborators, 2025; Grams et al., 2023). Because early CKD is often asymptomatic, it commonly goes unrecognised until declines in eGFR and/or rises in albuminuria are detectable (Stevens et al., 2024; Levey et al., 2022). Current frameworks, including KDIGO, stage CKD by GFR categories and markers of kidney damage—especially albuminuria—to inform prognosis and management (Stevens et al., 2024; Levey et al., 2022; Lameire et al., 2021). Although these measures have reshaped surveillance and care, they capture only part of the biological heterogeneity underlying early kidney injury across etiologies. In this mini-review, we use the term ‘early-stage CKD’ to denote individuals in KDIGO G1–G2 and selected G3a categories who have preserved or mildly reduced eGFR but A1–A2 albuminuria and/or other evidence of kidney damage, including normoalbuminuric phenotypes with structural, imaging or molecular signs of kidney injury.

Albuminuria is a robust predictor of kidney failure, cardiovascular events and all-cause mortality, and its reduction is an established therapeutic target in CKD (Levey et al., 2022; Appel et al., 2023). However, urinary albumin excretion is influenced by haemodynamic, metabolic and inflammatory factors, exhibits considerable intra-individual variability and may remain within the normal range in patients with predominant tubulointerstitial or vascular lesions (Claudel and Waikar, 2024; Matsuda et al., 2024; Buchwinkler et al., 2025; Aslan et al., 2025; Chagnac and Friedman, 2024; Canki et al., 2024; D’Marco et al., 2022). As a consequence, many individuals at high risk of progressive CKD are identified only after persistent albuminuria or measurable GFR decline has developed, which restricts the opportunity for timely, disease-modifying intervention (Stevens et al., 2024; Siwy et al., 2021; Villalvazo et al., 2025; Suo et al., 2025). These limitations have prompted intensive efforts to discover novel biomarkers that reflect defined pathophysiological processes, including tubular stress, inflammation, fibrosis and metabolic reprogramming, and that provide incremental value beyond albuminuria and estimated GFR for early detection and risk stratification, ideally improving model calibration, discrimination and risk reclassification in ways that meaningfully alter clinical decision-making (Siwy et al., 2021; Canki et al., 2024).

High-throughput omics now enables systematic profiling of the genome, epigenome, transcriptome, proteome, and metabolome in blood, urine, and kidney tissue, providing a finer-grained view of early kidney injury (Saliba et al., 2024; Liu X. et al., 2024; Rinschen and Saez-Rodriguez, 2021). In CKD and key etiologies such as diabetic kidney disease, proteomic and metabolomic studies have identified peptide and metabolite panels that predict incident albuminuria or faster GFR decline and, in some settings, discriminate early-stage CKD from healthy controls with high accuracy (Liu et al., 2021; Wen et al., 2022; Dubin et al., 2023; Suo et al., 2025; Ye et al., 2024). Integrative multi-omics studies further demonstrate that combining complementary molecular layers refines prognostic models for CKD trajectories and reveals mechanistic networks and candidate therapeutic targets that are not apparent from single-omics analyses (Alakwaa et al., 2025; Si et al., 2024; Liu X. et al., 2024; Li C. et al., 2021). Within this context, the shift from an albuminuria-centric paradigm towards composite multi-omics signatures offers an opportunity to redefine early-stage CKD in mechanistic terms, enabling earlier diagnosis, improved risk stratification and rational prioritisation of emerging drug targets for disease modification.

## 2 Pathophysiology of early-stage chronic kidney disease

Even in the earliest stages defined by modest albuminuria and preserved eGFR, chronic kidney disease reflects the interaction of systemic haemodynamic and metabolic stress with intrinsic renal responses that together initiate glomerular, tubular, interstitial and microvascular injury (Huang et al., 2023; Sohail et al., 2026; Levey et al., 2022; Li S. et al., 2021). These responses include glomerular hyperfiltration and intraglomerular hypertension in remnant nephrons, activation of the renin–angiotensin–aldosterone system, altered phosphate–FGF23–klotho signalling and low-grade inflammation (Stevens et al., 2024; Levey et al., 2022; Li S. et al., 2021), which together promote structural remodelling long before overt loss of kidney function is apparent.

**FIGURE 1:**
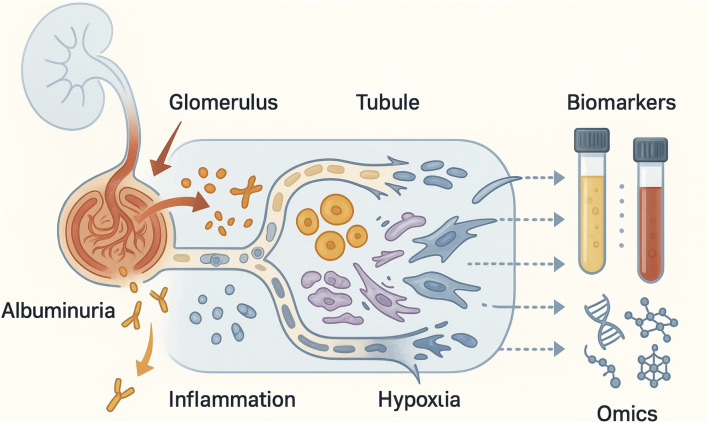
From glomerular albumin leakage to multi-omics biomarkers in early-stage chronic kidney disease.

As shown in Figure 1, at the glomerular level, increased transcapillary pressure and shear stress contribute to endothelial dysfunction, podocyte injury and expansion of the mesangial matrix (Levey et al., 2022; Li S. et al., 2021). Podocyte foot process effacement, loss of slit diaphragm integrity and disruption of the glomerular endothelial glycocalyx increase the permeability of the filtration barrier to albumin and other macromolecules, giving rise to microalbuminuria as an early clinical manifestation (Levey et al., 2022). Concurrently, activation of intraglomerular inflammatory and profibrotic pathways, including transforming growth factor-β (TGF-β) and angiotensin II–dependent signalling, promotes segmental glomerulosclerosis and lays the groundwork for progressive nephron loss (Huang et al., 2023; Levey et al., 2022).

Filtered albumin, lipids and glucose are reabsorbed by proximal tubular cells, where they can trigger oxidative stress, endoplasmic reticulum stress and activation of proinflammatory pathways, resulting in the production of chemokines and cytokines such as monocyte chemoattractant protein-1 and interleukin-6 (Huang et al., 2023; Zhang et al., 2021). These signals recruit monocytes, macrophages and lymphocytes to the interstitium and drive activation of fibroblasts and pericytes, leading to excess deposition of extracellular matrix and tubulointerstitial fibrosis, the final common pathway of progressive CKD (Huang et al., 2023; Sohail et al., 2026). Even in early-stage disease, experimental and clinical data show that tubular stress biomarkers and fibrosis-associated molecules are detectable in urine and tissue and are associated with subsequent decline in eGFR (Canki et al., 2024; Amatruda et al., 2024).

Microvascular injury and capillary rarefaction are integral components of this process. Structural and functional abnormalities of the renal microcirculation, including endothelial dysfunction, impaired nitric oxide bioavailability and enhanced vasoconstrictor tone, reduce cortical and medullary perfusion and create a hypoxic microenvironment (Li S. et al., 2021; Kannenkeril et al., 2021). Hypoxia stabilizes hypoxia-inducible factors and amplifies fibrogenic signalling, further coupling vascular damage to tubular atrophy and interstitial scarring (Habas et al., 2023; Naas et al., 2023; Zhang et al., 2021). As shown in Table 1, early-stage CKD can therefore be conceptualised as a set of overlapping injury axes—glomerular haemodynamic stress, tubular protein and nutrient overload, inflammatory and fibrotic remodelling, and microvascular dysfunction—that together shape the spatial pattern of kidney damage and its systemic consequences.

**TABLE 1:** Principal pathophysiological axes in early-stage chronic kidney disease and their relevance to biomarker discovery.

Pathophysiological axis	Predominant renal compartment	Representative mechanisms and molecular features	Potential biomarker domains
Glomerular haemodynamic stress	Glomerular capillary tuft, podocyte	Hyperfiltration, intraglomerular hypertension, podocyte cytoskeletal injury, altered slit diaphragm proteins (e.g., nephrin, podocin), mesangial expansion	Albuminuria pattern, urinary podocyte-derived proteins, circulating endothelial injury markers
Tubular protein and nutrient overload	Proximal tubule epithelium	Endocytic overload, oxidative and ER stress, activation of proinflammatory and profibrotic signalling, tubular epithelial dedifferentiation	Urinary tubular enzymes and transport proteins, chemokines, injury-associated transcripts and peptides
Interstitial inflammation and fibrosis	Interstitium, peritubular space	Recruitment of immune cells, activation of fibroblasts and pericytes, excess matrix deposition, myofibroblast persistence	Urinary and plasma extracellular matrix fragments, profibrotic growth factors, stromal cell–derived transcripts
Microvascular dysfunction and hypoxia	Peritubular capillary network	Endothelial dysfunction, capillary rarefaction, impaired vasodilatory responses, tissue hypoxia, altered FGF23–klotho–phosphate axis	Circulating and urinary endothelial injury markers, angiogenic factors, hypoxia-responsive metabolites

These structural and cellular perturbations are accompanied by coordinated alterations across genomic, epigenomic, transcriptomic, proteomic and metabolomic layers. In diabetic and non-diabetic CKD, omics and multi-omics analyses increasingly reveal early enrichment of pathways related to innate immunity, complement activation, extracellular matrix remodelling, mitochondrial dysfunction and disordered energy metabolism in kidney tissue, blood and urine (Liu et al., 2021; Alakwaa et al., 2025; Md Dom et al., 2025; Dubin et al., 2023; Li C. et al., 2021). Such network-level changes can precede overt loss of GFR and may persist after the inciting stimulus has resolved, supporting the concept of maladaptive repair and “primed” kidney injury after acute insults (Lameire et al., 2021). Recurrent clinically overt or subclinical episodes of acute kidney injury with incomplete repair can therefore drive a trajectory into ‘early CKD’, such that many early-stage molecular signals reflect the cumulative footprint of repeated injury superimposed on relatively preserved filtration rather than slow, primary chronic disease alone. Early-stage CKD is best understood as a dynamic systems-level disorder in which compartment-specific injury responses and systemic mediators interact to determine both clinical manifestations, including albuminuria.

## 3 Limitations of albuminuria and conventional markers for early kidney damage

Albuminuria and estimated glomerular filtration rate remain the main clinical markers for chronic kidney disease detection and staging, but their ability to detect early kidney damage is limited. Albuminuria predominantly reflects injury to the glomerular filtration barrier and only indirectly mirrors downstream tubular, interstitial and vascular pathology (Levey et al., 2022; Canki et al., 2024). Clinical and pathological observations in diabetic and non-diabetic kidney disease demonstrate heterogeneity of structural lesions, with substantial tubulointerstitial and/or vascular injury present in some individuals with normoalbuminuria or low-grade albuminuria (D’Marco et al., 2022). In hypertensive nephrosclerosis and ischemic nephropathy, extensive arteriolar and interstitial damage may occur with modest or absent albuminuria, indicating that reliance on albuminuria alone can underestimate the burden of early kidney injury in specific etiologies (Canki et al., 2024).

Measurement variability limits albuminuria as an early risk marker. Urinary albumin excretion and ACR vary with posture, activity, diet, acute glycaemic and blood pressure changes, intercurrent illness and other short-term influences (Beernink et al., 2025; Claudel and Waikar, 2024; Matsuda et al., 2024; Buchwinkler et al., 2025; Aslan et al., 2025; Chagnac and Friedman, 2024; Canki et al., 2024). Within-person coefficients of variation for urine albumin often exceed 20%, obscuring true progression in patients (Claudel and Waikar, 2024; Matsuda et al., 2024; Buchwinkler et al., 2025; Aslan et al., 2025). Hence first-morning ACR is more reliable than random spot urine for diagnosing and monitoring microalbuminuria (Witte et al., 2009). This instability necessitates repeat testing, reduces staging precision, and weakens albuminuria as a surrogate endpoint when antiproteinuric effects are small or uncoupled from structural preservation. Similar issues likely affect omics markers, for which within-person variability, short-term stability and illness sensitivity remain poorly characterised; robust assessment of short-to medium-term variation is required before fixed omics thresholds guide individual care.

Conventional functional markers show analogous limitations. Serum creatinine depends on muscle mass, diet, tubular secretion and assay interference, and creatinine-based equations are least reliable at higher GFR, where early injury is most amenable to intervention, so the choice of filtration marker—creatinine, cystatin C or combined equations—can alter risk classification and perceived added value of omics signals, particularly when reference eGFR is noisy or biased by muscle mass or protein intake (Levey et al., 2022). Compensatory hyperfiltration can maintain normal-range eGFR despite nephron loss or segmental scarring, allowing structural damage to accumulate while eGFR and albuminuria remain within guideline thresholds (Levey et al., 2022; Lameire et al., 2021). Tubular injury markers (KIM-1, NGAL, L-FABP, MCP-1, uromodulin) show modest, often non-independent associations with CKD progression and confer limited gains in risk prediction beyond eGFR and albuminuria (Canki et al., 2024; Amatruda et al., 2024) and, in many proteomic platforms, are incorporated as individual features within composite multi-omics scores rather than applied as stand-alone replacements for albuminuria or eGFR (Schlosser et al., 2023). Beyond analytical noise, sex hormones, body composition, diabetes, dyslipidaemia, low-grade inflammation, SGLT2 inhibitors, renin–angiotensin–aldosterone blockade and infections or cardiovascular events reshape proteomic and metabolomic profiles and thus biomarker distributions and cutoffs. Omics signatures may therefore index systemic cardiometabolic risk rather than kidney-specific injury, necessitating careful covariate adjustment when deriving, validating and thresholding candidate biomarkers.

## 4 Emerging omics-based biomarkers for early-stage chronic kidney disease

High-throughput omics platforms have generated biomarkers that capture early molecular perturbations in chronic kidney disease before measurable loss of glomerular filtration rate (Dharmarathne et al., 2024). Proteomic, metabolomic, transcriptomic and epigenomic assays in blood, urine and kidney tissue reveal signatures linked to tubular injury, inflammation, fibrosis and mitochondrial dysfunction and are evaluated as tools for earlier detection and refined risk stratification (Liu X. et al., 2024; Lopes et al., 2025). Each biospecimen matrix, however, has distinct physiological and pre-analytical constraints. Urine-based assays are strongly influenced by hydration status, collection timing, recent exercise and urinary tract infection; plasma and serum measurements are affected by fasting state and circadian rhythms; and tissue-based omics depend on biopsy site, regional heterogeneity and warm and cold ischaemia times between devascularisation and freezing.

Among proteomic approaches, urinary peptide panels derived from capillary electrophoresis–mass spectrometry, most prominently the CKD273 classifier, predict incident stage 3 chronic kidney disease and albuminuria progression in diabetes and mixed-etiology cohorts and provide added prognostic value beyond estimated glomerular filtration rate and albuminuria (Verbeke et al., 2021; Pontillo and Mischak, 2017; Pontillo et al., 2017; Suo et al., 2025). Prospective evaluation in the PRIORITY study showed that a high-risk CKD273 score in normoalbuminuric type 2 diabetes identified individuals at increased risk of developing microalbuminuria, supporting its use to enrich early trials (Suo et al., 2025). Other urinary proteomic and integrative omics analyses have also nominated candidate proteins linked to extracellular matrix remodelling, inflammation and tubular stress in early diabetic kidney disease (Li et al., 2025; Swaminathan et al., 2023). Urinary and extracellular vesicle (EV)-based profiling has further yielded candidate markers that distinguish early diabetic kidney disease from controls and correlate with disease severity (Canki et al., 2024; Swaminathan et al., 2023). Because EVs are actively secreted by tubular epithelial and other renal cells and carry concentrated protein and microRNA cargo, they may better reflect ongoing intrarenal cellular signalling than bulk urine or plasma (He et al., 2024; Koide et al., 2023; Canki et al., 2024; Erdbrügger and Le, 2016), positioning EV-based assays as an intermediate step between purely systemic biomarkers and invasive tissue sampling on the translational spectrum.

Metabolomic profiling provides an additional layer of information on disordered intermediary metabolism in early-stage disease. Untargeted plasma and urine metabolomics have identified alterations in amino acids, acylcarnitines, tricarboxylic acid cycle intermediates and uremic solutes that associate with baseline kidney function and predict subsequent eGFR decline (Wen et al., 2022; Qiu et al., 2023; Ragi and Sharma, 2024; Luo et al., 2024; Hosseinkhani et al., 2022; Sabanayagam et al., 2023). In addition, specific metabolites such as endogenous adenine have been linked to kidney injury in experimental models and to diabetic kidney disease risk in patients, illustrating how metabolite biomarkers can nominate mechanistic pathways for intervention (Sharma et al., 2023; Ragi and Sharma, 2024; Drexler and Fornoni, 2023). Integration of metabolite and renal transcriptomic data indicates that many signatures reflect impaired energy metabolism and oxidative stress, linking circulating metabolic fingerprints to intrarenal processes (Liu et al., 2021; Wen et al., 2022; Liu X. et al., 2024).

Emerging transcriptomic, epigenomic and non-coding RNA biomarkers further extend mechanistically anchored early detection. Transcriptome-wide profiling of kidney biopsies and urinary sediment has defined gene-expression modules related to innate immunity, fibrosis and tubular dedifferentiation that stratify progression risk (Ju et al., 2015). Epigenetic studies report differential DNA methylation at loci involved in inflammation, fibrosis and lipid metabolism and have derived blood-based methylation risk scores that associate with prevalent or incident CKD (Lecamwasam et al., 2021; Marchiori et al., 2025; Jones et al., 2024; Sagy et al., 2024; Suo et al., 2025; Kremer et al., 2022). Profiling of circulating and urinary microRNAs and long non-coding RNAs has identified species associated with diabetic kidney disease onset and tubulointerstitial fibrosis (Liu Z. et al., 2024; Koide et al., 2023; Hüttenhofer and Mayer, 2017; Coellar et al., 2021). These omics-based biomarkers offer complementary views of early kidney injury and provide a foundation for composite molecular signatures with improved sensitivity and specificity for early-stage chronic kidney disease.

## 5 Multi-omics signatures for risk stratification and patient subtyping

Multi-omics approaches integrate genomic, transcriptomic, proteomic, metabolomic and, in some studies, epigenomic or spatial data from blood, urine and kidney tissue (Saliba et al., 2024; Hodgin et al., 2024; Liu X. et al., 2024; Rinschen and Saez-Rodriguez, 2021; Benito et al., 2024). By jointly analysing these layers with clinical variables, recent studies have derived composite molecular signatures that better reflect key biological processes in early chronic kidney disease and improve prediction of renal outcomes compared with creatinine, estimated glomerular filtration rate and albuminuria alone (Liu et al., 2021; Alakwaa et al., 2025; Dubin et al., 2023; Li C. et al., 2021; Dharmarathne et al., 2024; Ye et al., 2024). Proteomic and metabolomic panels summarise concurrent alterations in extracellular matrix remodelling, inflammation and intermediary metabolism, and can be combined with genetic or transcriptomic markers to construct multivariate risk scores for incident chronic kidney disease or accelerated decline in glomerular filtration rate (Liu et al., 2021; Wen et al., 2022; Dubin et al., 2023; Si et al., 2024). However, most multi-omics signatures to date have been derived in cohorts enriched for diabetic kidney disease, and their transportability to other common aetiologies such as hypertensive nephrosclerosis, IgA nephropathy or autosomal dominant polycystic kidney disease (ADPKD) remains uncertain (Aydogan Balaban et al., 2025). Differences in the dominant injury compartments, immunopathology and background therapies mean that a proteometabolomic pattern strongly predictive in diabetes may be only weakly informative, or even misleading, when applied to these non-diabetic phenotypes. Representative examples of omics-derived and multi-omics signatures that provide diagnostic and/or prognostic information beyond conventional KDIGO classification are summarised in Table 2.

**TABLE 2:** Selected omics and multi-omics biomarkers/Signatures that provide diagnostic or prognostic value beyond conventional KDIGO classification (eGFR and albuminuria).

Study/Cohort (reference)	Omics modality and specimen/Signature	Population/Early-CKD context	Key result beyond eGFR/Albuminuria (KDIGO)
PRIORITY study (Suo et al., 2025)	Urinary proteomics (CE–MS); CKD273 classifier	Normoalbuminuric T2D with preserved eGFR	High-risk CKD273 identified individuals more likely to develop microalbuminuria, enabling enrichment beyond baseline UACR/eGFR.
CKD273 stage-3 prediction (Pontillo and Mischak, 2017; Pontillo et al., 2017)	Urinary peptide panel (CKD273)	Diabetes and mixed-etiology cohorts	Predicted incident CKD stage 3 and progression independent of baseline eGFR and albuminuria
CKD273 & CV outcomes (Verbeke et al., 2021)	Urinary peptide panel (CKD273)	Established CKD	Associated with cardiovascular outcomes in CKD beyond eGFR and albuminuria, highlighting complementary systemic risk capture
Large-scale proteomics (Dubin et al., 2023; Ye et al., 2024)	Plasma proteomics panels	Diabetes/CKD cohorts	Proteomic signatures improved prediction of CKD incidence/progression compared with clinical models based on eGFR and albuminuria
Serum integrative omics (Liu et al., 2021)	Integrated serum proteomics/metabolomics	Diabetic kidney disease	Multi-omics patterns associated with DKD severity/progression and highlighted pathways not captured by eGFR/albuminuria alone
Metabolomics and adenine (Wen et al., 2022; Sharma et al., 2023; Ragi and Sharma, 2024; Drexler and Fornoni, 2023)	Plasma/serum metabolomics; candidate metabolites (e.g., adenine)	CKD progression and diabetic models	Metabolite signatures predicted eGFR decline and nominated mechanistic targets (adenine-related pathways) beyond conventional markers
Kidney molecular categories (Reznichenko et al., 2024; Li et al., 2021b)	Kidney tissue transcriptomics with urine translation	Multi-etiology CKD	Molecular categories showed distinct progression rates; urine classifiers enabled non-invasive assignment beyond conventional clinical labels

Urinary proteomics provides a central component of these multi-marker signatures. Pontillo et al. reported that the CKD273 classifier, a panel of 273 urinary peptides enriched for collagen fragments and proteins associated with tubular stress and fibrosis, predicts development of stage 3 chronic kidney disease beyond baseline albuminuria and estimated glomerular filtration rate in high-risk cohorts (Pontillo and Mischak, 2017; Pontillo et al., 2017). In normoalbuminuric type 2 diabetes, the PRIORITY study showed that higher CKD273 scores identify individuals with increased probability of progression to microalbuminuria, enabling enrichment of early-intervention trials with patients at greatest risk despite preserved conventional markers (Suo et al., 2025). In established CKD, CKD273 has also been associated with cardiovascular outcomes, highlighting that urinary proteomic signatures may capture systemic risk that complements kidney-specific risk stratification (Verbeke et al., 2021; Tofte et al., 2020). These findings indicate that integrating numerous low-abundance peptide signals into a single continuous score refines risk stratification in individuals who would otherwise be classified as low risk by conventional markers.

Multi-omics studies of kidney biopsy tissue extend this approach from risk prediction towards mechanistic patient subtyping. Reznichenko et al. performed an unbiased analysis of cortical transcriptomes from several chronic kidney disease cohorts and identified reproducible molecular categories that spanned conventional aetiological and histopathological labels and were associated with distinct pathway activation patterns and significantly different rates of progression to kidney failure (Reznichenko et al., 2024; Li C. et al., 2021). Importantly, integration with urine proteomic data enabled development of a non-invasive classifier capable of distinguishing the most aggressive molecular subgroup, supporting the feasibility of translating tissue-based signatures into accessible assays for molecular categorisation (Li C. et al., 2021). Complementary multi-omic analyses of human kidney tissue, combining bulk and spatial transcriptomics with proteomics and metabolomics, further delineate compartment-specific injury pathways and candidate therapeutic targets that align with these molecular categories (Asowata et al., 2024).

## 6 From signatures to therapies: emerging drug targets and therapeutic strategies

Multi-omics signatures not only refine risk stratification but also delineate druggable mechanisms in early-stage chronic kidney disease. Systems biology frameworks that integrate transcriptomic, proteomic and metabolomic data can identify co-regulated disease modules, map them to signalling pathways and overlay these modules with genetic instruments and drug–target databases (Villalvazo et al., 2025; Si et al., 2024; Schlosser, 2023). In CKD, such approaches have been used to prioritise candidate therapeutic targets by combining kidney tissue and circulating omics layers with Mendelian randomisation and network analyses, yielding panels of proteins that cluster in immune, complement and extracellular-matrix pathways and differ across aetiologies and clinical subtypes (Xu et al., 2024; Md Dom et al., 2025; Dubin et al., 2023; Si et al., 2024; Liu et al., 2023; Cuarental et al., 2023). Many of these molecules are best viewed as biomarkers of underlying pathway activity or nodal network behaviour, and only a subset are realistically tractable as direct drug targets because of issues such as ubiquitous expression, redundancy, compensatory pathways and potential on-target toxicity. These results provide a rational basis for mechanism-based intervention in biologically defined patient groups rather than solely in albuminuria- or eGFR-based categories.

The mechanistic pathways highlighted by omics analyses converge on both established and emerging drug classes. Haemodynamic–metabolic modules implicating tubular sodium–glucose transport, mineralocorticoid receptor signalling and endothelin pathways are concordant with the renoprotective effects of sodium–glucose cotransporter-2 inhibitors, non-steroidal mineralocorticoid receptor antagonists and endothelin A receptor antagonists (Heerspink et al., 2021; Rossing et al., 2022; Heerspink et al., 2020; Bakris et al., 2020; Agarwal et al., 2025; Ma et al., 2025; Tian et al., 2025). Multi-omics and imaging mass cytometry studies further emphasise inflammatory tubular cell states and fibroblast–immune cell niches as central nodes, and experimental targeting of senescent tubular cells with senolytics can ameliorate tubulointerstitial fibrosis, illustrating how cellular signatures can nominate tractable therapeutic strategies (Asowata et al., 2024; Li C. et al., 2021; Mylonas and Ferenbahc, 2024; Wang, 2021; Chen et al., 2024). Complement-enriched proteomic and genetic signatures, together with clinical data, support complement components as additional targets, and complement inhibitors are being evaluated in proteinuric glomerulopathies characterised by complement dysregulation (Md Dom et al., 2025; Bomback et al., 2022; Wooden et al., 2023; Shao et al., 2025).

Translation of signatures into therapies requires integration of molecular information into trial design and clinical decision-making. Kidney tissue transcriptomic categories of CKD, defined by distinct patterns of inflammatory, metabolic and fibrotic pathway activation and linked to non-invasive urine classifiers, provide a framework to enrich early-stage trials with patients who harbour the molecular processes targeted by a given agent and to monitor on-target pathway modulation (Li C. et al., 2021; El-Achkar et al., 2024). Multi-omics readouts can also function as pharmacodynamic biomarkers that quantify partial reversal of disease modules under treatment, even when changes in albuminuria or eGFR are small (El-Achkar et al., 2024). These strategies support development of therapeutic algorithms that combine mechanism-based drug selection with molecularly defined CKD subgroups, aiming to achieve durable disease modification at a stage when kidney structure remains at least partially reversible.

## 7 Summary and future perspective

Multi-omics technologies have expanded the biomarker repertoire for early-stage chronic kidney disease—here referring largely to KDIGO G1–G2 and selected G3a categories with preserved eGFR but evidence of kidney damage, including normoalbuminuric phenotypes—beyond albuminuria and estimated glomerular filtration rate by capturing pathophysiological processes such as tubular stress, inflammation, fibrosis and metabolic dysregulation at high resolution (Saliba et al., 2024; Siwy et al., 2021; Canki et al., 2024; Liu X. et al., 2024; Rroji and Spasovski, 2025). Urinary proteomic classifiers and blood-based proteomic/metabolomic signatures identify individuals who are at increased risk of incident albuminuria and accelerated kidney function decline despite preserved conventional markers, and improve risk discrimination when added to clinical models (Verbeke et al., 2021; Liu et al., 2021; Wen et al., 2022; Dubin et al., 2023; Suo et al., 2025; Ye et al., 2024). These data, together with integrative analyses of multi-omics panels, support the concept that composite molecular signatures can resolve heterogeneous trajectories of early kidney damage and delineate biologically coherent patient subgroups that differ in prognosis and (potentially) therapeutic responsiveness (Alakwaa et al., 2025; Li C. et al., 2021; Dharmarathne et al., 2024). At the same time, recent commentaries emphasise that novel biomarkers should be interpreted within a pathophysiological framework and anchored to defined mechanisms of injury to ensure clinical relevance (Siwy et al., 2021).

Future research needs to consolidate these advances into robust tools for precision prevention and therapy in early-stage chronic kidney disease. Large, prospectively phenotyped cohorts with repeated biosampling and adjudicated renal and cardiovascular outcomes across diverse aetiologies and ancestries are required to validate candidate multi-omics signatures, quantify their incremental value over albuminuria and eGFR in terms of improved calibration, discrimination, risk reclassification and clinical net benefit, and assess analytical, pre-analytical and biological sources of variability (El-Achkar et al., 2024). Standardised pipelines for biospecimen handling, data generation, quality control and integration, together with transparent reporting and external validation, will be essential to facilitate comparison across studies, support regulatory qualification of multi-omics-derived risk scores, and detect/correct for batch effects and platform changes that can shift absolute measurements and thereby invalidate fixed biomarker cutoffs over time (Schlosser et al., 2023; El-Achkar et al., 2024). Increasing use of advanced machine-learning algorithms must be coupled with biologically informed feature selection and independent replication to avoid overfitting and to maintain interpretability (Dharmarathne et al., 2024; Gulamali et al., 2022). Finally, pragmatic implementation studies and health-economic analyses are needed to determine how multi-omics testing can be integrated with electronic health records and decision-support systems in a cost-effective and equitable manner (Yeo et al., 2024; Agathangelou et al., 2025).

Translation into clinical practice will depend on demonstration that multi-omics-guided strategies enable earlier initiation of renoprotective therapies, more efficient trial enrichment and selection of mechanism-based interventions for molecularly defined subgroups, leading to improved patient-centred outcomes (McDonnell et al., 2025). Ultimately, convergence of molecular biomarkers, precise clinical phenotyping and targeted therapeutic strategies has the potential to redefine early-stage chronic kidney disease in mechanistic terms and to support a shift from late detection and uniform treatment towards timely, individualised disease modification.
